# Complex Responses to Climate Warming of Arctic‐Alpine Plant Populations From Different Geographic Provenance

**DOI:** 10.1002/ece3.71146

**Published:** 2025-03-19

**Authors:** Lisa Brancaleoni, Renato Gerdol, Andrea Mondoni, Simone Orsenigo, Lisa Scramoncin, Carla Lambertini, Chiara Cianferoni, Thomas Abeli

**Affiliations:** ^1^ Department of Environmental and Prevention Sciences University of Ferrara Ferrara Italy; ^2^ Department of Earth and Environmental Sciences University of Pavia Pavia Italy; ^3^ National Biodiversity Future Center (NBFC) Palermo Italy; ^4^ Department of Biosciences University of Milan Milan Italy

**Keywords:** controlled culture, core and marginal areas, distribution range, molecular analysis, phylogeographic variation, populations, provenance test, *Viscaria alpina*, water use efficiency

## Abstract

The distribution of ‘cold‐adapted’ plant species is expected to undergo severe range loss in the near future. Species distribution models predicting species' future distribution often do not integrate species ability to respond to environmental factors through genetic traits or phenotypic plasticity. This especially applies to arctic‐alpine species whose present‐day range is strongly fragmented because of the cyclic vicissitudes they experienced during the Ice Age. We cultivated plants from four European populations of the arctic‐alpine species *Viscaria alpina* from different geographic provenances. Two of the populations were from northern high‐latitude regions in Scandinavia; the remaining two populations were from southern mid‐latitude mountains. In both areas, one population was from a colder site and the other from a warmer site. We cultivated the plants in controlled thermal conditions with two treatments, one mimicking temperature conditions at the warmest site and the other adding 2 day‐temperature peaks mimicking short‐term heat waves. At the end of the experiment, we measured growth in length and mortality of all plants along with a set of ecophysiological variables. We also assessed phylogeographic variation in the four populations based on plastid‐DNA sequences. The plants from northern provenances grew more than those from the southern provenances. The plants of all populations performed overall well, in terms of growth rate and ecophysiology, under the heat spell, with the plants of the Swedish population exhibiting the highest phenotypic plasticity. Such a pattern was associated with the highest genetic variation in the Swedish population. Mortality of the plants cultivated under warm temperatures was overall low, but mortality strongly increased in the plants exposed to the heat spell. We conclude that plants of *V. alpina* populations from different geographic provenances are generally able to cope with scenarios resulting from global warming, but drought hampers resilience to heat waves through increased mortality.

## Graphical abstract

The distribution of ‘cold‐adapted’ plant species is expected to undergo severe range loss in the near future. Arctic species are predicted to experience range contraction at their southern trailing‐edge locations. The distribution range of alpine species is expected to shrink at their lower‐elevation trailing‐edge locations. For arctic‐alpine species, it is conceptually difficult to evaluate the geographic location of trailing‐edge vs. leading‐edge populations because arctic‐alpine species underwent multiple re‐colonization events during the Pleistocene. We cultivated plants from four European populations of the arctic‐alpine species *Viscaria alpina* from different geographic provenances, two populations from high‐latitude regions and two populations from southern mid‐latitude mountains. The plants experienced two treatments, one mimicking temperature conditions at the warmest provenance site and the other adding 2 day‐temperature peaks mimicking short‐term heat waves. The plants of all populations performed overall well under both treatments, with the plants of the Swedish population exhibiting the highest phenotypic plasticity in association with high genetic variation. Mortality of the plants cultivated under warm temperatures was overall low, but mortality strongly increased in the plants exposed to the heat spell because of a detrimental drought effect.

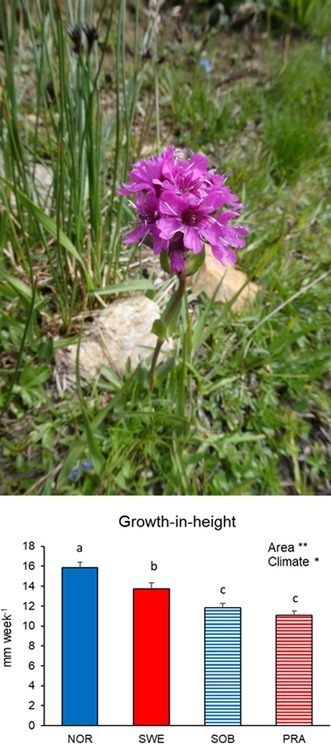

## 1 Introduction

Extinction and local extirpation with consequent species turnover are expected to occur in cold environments because of reduced habitat suitability for resident species and improved conditions for low elevation or low latitude migrants (Gottfried et al. 2012; Pauli et al. 2012; Porro et al. 2019). Hence, the distribution of ‘cold‐adapted’ species is projected to undergo severe range loss in the near future (Steinbauer et al. 2020). Arctic species are predicted to experience range contraction at their southern trailing‐range locations while the distribution range of northern, leading‐edge populations may even expand beyond their historical cold border by migrating poleward (Hickling et al. 2006). For alpine species, the distribution range of lower‐elevation trailing‐edge populations is expected to shrink, unless these peripheral plants possess full or partial adaptations to expected climate warming scenarios (Dullinger et al. 2012; Angert et al. 2020). Cold‐adapted alpine species are also predicted to shift their ranges towards colder locations, i.e., towards higher elevations (Lenoir et al. 2008) or in micro‐topographic niches (Körner and Hiltbrunner 2021). Distribution shifts triggered by climate change are projected using correlational bioclimate envelope models, which can overestimate species losses because key aspects such as demographic dynamics, plant plasticity, etc. are ignored by most models (Randin et al. 2009; Niskanen et al. 2017; Casazza et al. 2021). Phenotypic plasticity, i.e., the responsiveness of plants to changing environments, consists in the capacity of a genotype to express different phenotypes under diverse environmental conditions (Kawecki and Ebert 2004; Garland and Kelly 2006; Leimu and Fischer 2008). If the performance of populations is correlated with environmental conditions at the provenance site, this can be interpreted as a test of local adaptation, although local adaptation may be marginal in certain species and environments (Macel et al. 2007; Beierkuhnlein et al. 2011). Peripheral trailing‐edge plant populations often host unique genetic traits when compared to core populations (Eckert et al. 2008; Mathiasen et al. 2021). Local adaptation may be particularly important at range limits where the selective pressure of climatic conditions on a species' population is usually stronger than in its range center and where genetic recombination may be limited due to geographic isolation (Choler et al. 2004; Kawecki 2008; Paul et al. 2011). It is less clear whether some of the unique genetic traits of peripheral populations may be useful to cope with climate change. Abeli et al. (2018) hypothesized that peripheral plant populations of cold‐adapted species may already be adapted to local warm conditions, especially in southern refugia. This seems to be supported by the long‐term stability of several trailing‐edge populations (Abeli et al. 2012; Mathiasen et al. 2021) and eventually by models in which demographic and genetic simulations are included (Cotto et al. 2017).

Results of common garden experiments suggest that genotypes from warmer sites in many cases have an advantage in terms of demographic traits compared to genotypes from colder sites when exposed to warming (Peterson et al. 2016; Bontrager and Angert 2019). For example, individuals of *Eucalyptus grandis* experiencing natural long‐term exposure to heat waves have a higher capacity to express protective proteins than individuals from populations with lower exposure to extreme weather events (Maher et al. 2019). Dickman et al. (2019) recorded adaptation to drought in seeds from peripheral low‐elevation populations of *Mimulus laciniatus* . On the other hand, there is increasing empirical evidence that plants can react to climate warming with mechanisms involving phenotypic plasticity (Schneider 2022). Plasticity allows plants to grow under variable conditions and to cope with environmental changes by immediate (plastic) responses. There is strong evidence that phenotypic plasticity is directly conditioned by phylogeographic variation (Eller et al. 2017). However, phenotypic plasticity can also derive from changes in gene expression controlled by epigenetic response, which confers plants the ability to respond in the short term to a rapidly changing environment (Nicotra et al. 2010; Pagliarani and Gambino 2019; Ashapkin et al. 2020). Furthermore, phenotypic plasticity can be triggered by the intrinsic ability of plants to acclimate to changing ecological conditions (Valdés et al. 2019). On the other hand, when environmental conditions exert strong selective pressures on organisms, like in ecologically/geographically peripheral populations, useful trait selection and stabilization may result in the loss of plasticity (Anstett et al. 2021; Usui et al. 2023). Whatever the mechanisms involved, phenotypic plasticity plays an important role in explaining the adaptation of cold‐climate plants to warming climate. For example, a high level of phenotypic variation buffers the alpine cliff plant *Heterotheca brandegeei* from the negative impacts of warming and drought in southern North America (Winkler et al. 2019). For arctic‐alpine plant species, it is more problematic to define where core areas and marginal areas are located within their highly fragmented distribution ranges. Therefore, it is conceptually difficult to evaluate the geographic location of trailing‐edge vs. leading‐edge populations of arctic‐alpine species that experienced multiple re‐colonization events during the climatic vicissitudes that occurred during the Pleistocene. Based on their complex colonization history, populations of arctic‐alpine species may reflect differing pathways of genetic differentiation, possibly untethered by latitude. Furthermore, the sites located at the southern margin of the present‐day distribution area do not always represent the warmest sites within the species' range (Abeli et al. 2015). This may constitute a mismatch between geographic and ecological marginality (Soule 1973). It is generally assumed that the distribution range of southern populations of arctic‐alpine species will shrink unless these peripheral plants possess full or partial adaptations to expected climate warming scenarios (Abeli et al. 2018; Dullinger et al. 2012). The distribution range of northern populations may even expand beyond their historical cold border. However, northward expansion will be precluded if warming negatively impacts the growth of plants from northern populations at cooler temperatures compared to plants from southern populations (DeMarche et al. 2018). Interestingly, a recent study predicted a stronger decline of arctic‐alpine species at the northern range margins than at southern locations (Niskanen et al. 2019).

**FIGURE 1:**
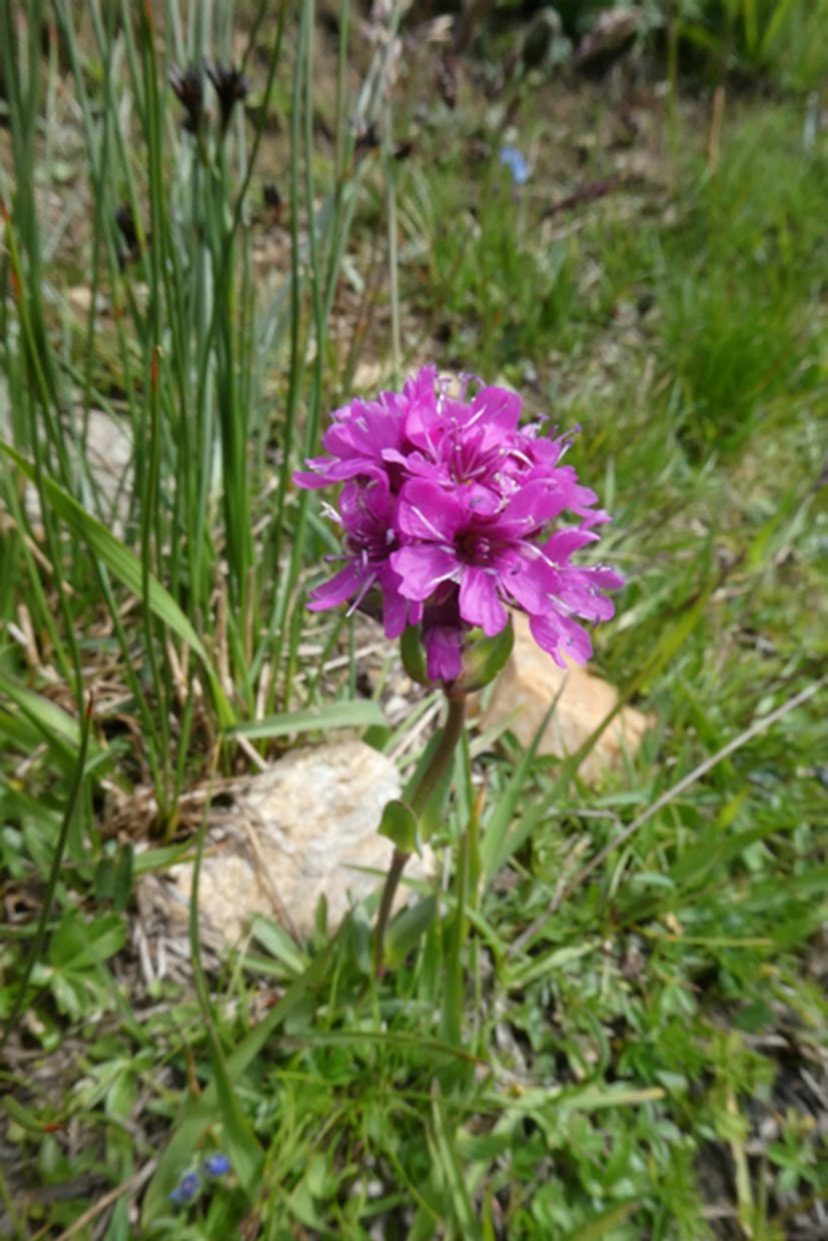
A flowering plant of *Viscaria alpina* at the Monte Sobretta site (Central Alps, Italy).

In this study, we performed a provenance test (Mátyás 1996) with the aim of analyzing the response of an arctic‐alpine species to a warming climate. We used the Alpine catchfly [ *Viscaria alpina* (L.) G. Don; Figure 1] as an excellent model to examine multi‐faceted responses of different populations to global warming. In fact, *V. alpina* has a broad distribution range that consists of a vast high‐latitude area extending from North America to eastern Eurasia and a set of smaller mid‐latitude mountain areas prevalently located in southern Europe (Nagy 2013). *V. alpina* exhibits relatively high phylogeographic variation among populations whose locations differ strongly in terms of climatic, altitudinal, and edaphic features (Haraldsen and Wesenberg 1993). Most importantly, both the southern and the northern populations of *V. alpina* span a wide thermal range with differences of up to 7°C–9°C in average mean annual temperature (Nagy 2013).

## 2 Material and Methods

### 2.1 Study Material and Experimental Design

**FIGURE 2:**
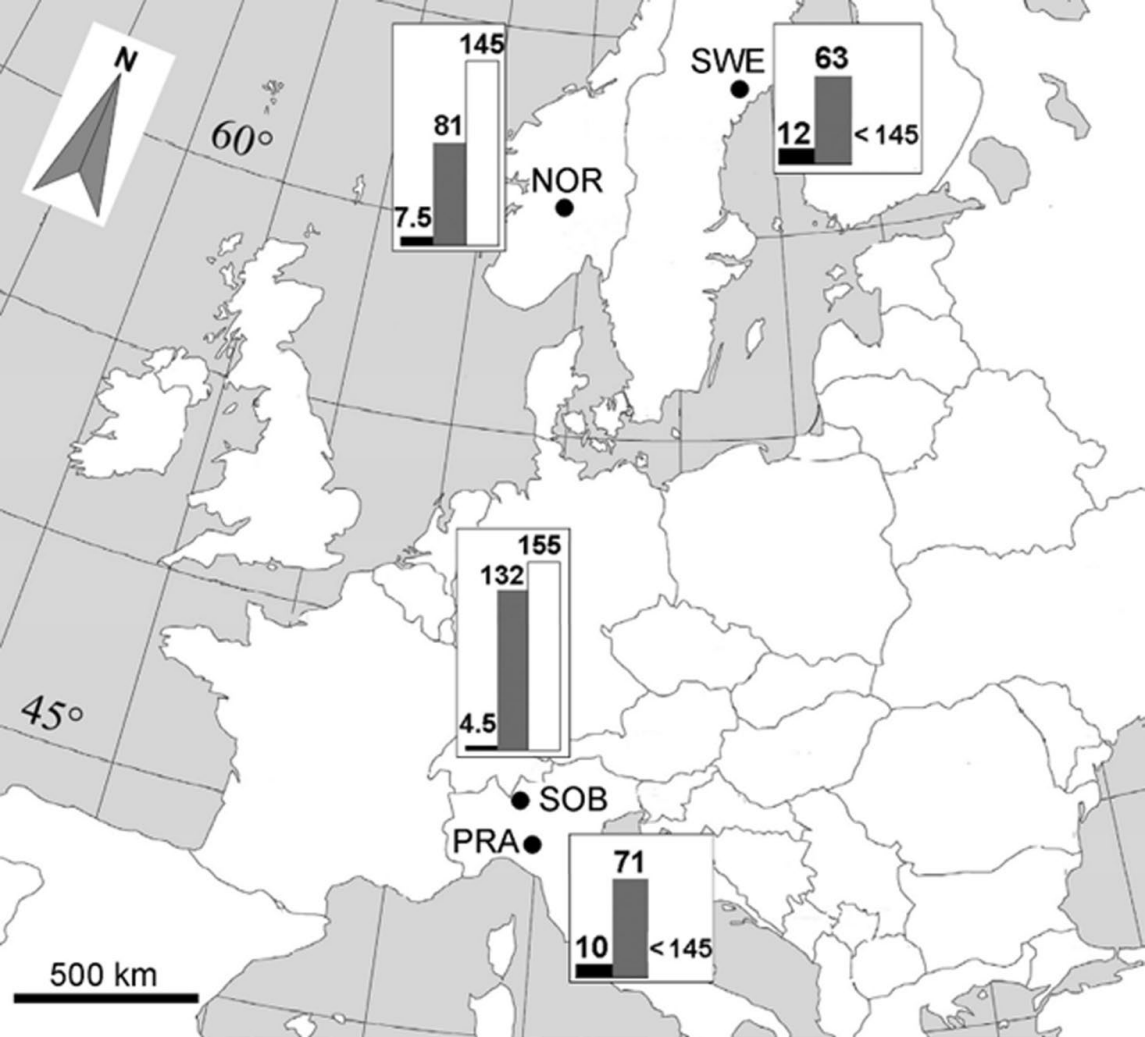
Geographic location of the *V. alpina* populations. NOR: Oppland, Norway (1070 m); SWE: Västerbotten, Sweden (230 m); SOB: Monte Sobretta, Italy (2700 m); PRA: Monte Prado, Italy (2040 m). For each site, the bars indicate mean temperature (as °C, black) and mean precipitation (as mm, gray) of the growing season (from June to August) and mean number of snow‐free days during the year. Reproduced from Abeli et al. (2015). Doi: 10.1007/s11284‐014‐1225‐3.

We selected four European populations, two from a northern area and two from a southern area. In both areas, one of the two populations stemmed from a colder site and the other from a warmer site, with a mean annual temperature difference of about 5°C between the two sites. We cultivated plants originated from seeds collected from the four populations at warm temperatures and assessed their responses in terms of morphological and ecophysiological traits based on phenotypic short‐term responses to high temperature. Furthermore, we cultivated a further set of plants under more extreme thermal conditions that simulated a short‐term heat spell mimicking heat wave conditions. Indeed, the frequency of heat waves is increasing in recent years, which results in compound effects of heat and drought (Mukherjee and Mishra 2021) besides the single effects of higher temperature. Experiments were conducted on seedlings, as this early life stage is highly sensitive to variation in climate and represents a major bottleneck to species recruitment, playing a key role in the distribution and dynamics of plant populations (Vázquez‐Ramírez and Venn 2021). The northern populations of *V. alpina* came from Scandinavia (Oppland, Norway and Västerbotten, Sweden; hereafter referred to as NOR and SWE, respectively; Figure 2), while the southern populations came from two Italian mountain regions (Monte Sobretta, Central Alps and Monte Prado, Northern Apennines; hereafter referred to as SOB and PRA, respectively; Figure 2). The four provenance sites differed in terms of climatic conditions besides latitude and corresponding location within the species' range: NOR and SOB were colder sites, while SWE and PRA were warmer sites (Abeli et al. 2015; Figure 2).

In summer 2012, seeds of *V. alpina* plants were collected at each site. The plants were cultivated at the Botanical Garden of the University of Pavia, at approximately 85 m above sea level, and produced seeds for the first time in 2015. Seeds of all populations were harvested and kept under standard seedbank conditions (−20°C after drying at 15%RH, 15°C) until used for cultivation in controlled conditions. Leaves of six to eight mature plants per population were harvested and dried in silica gel for subsequent molecular analyses.

**FIGURE 3:**
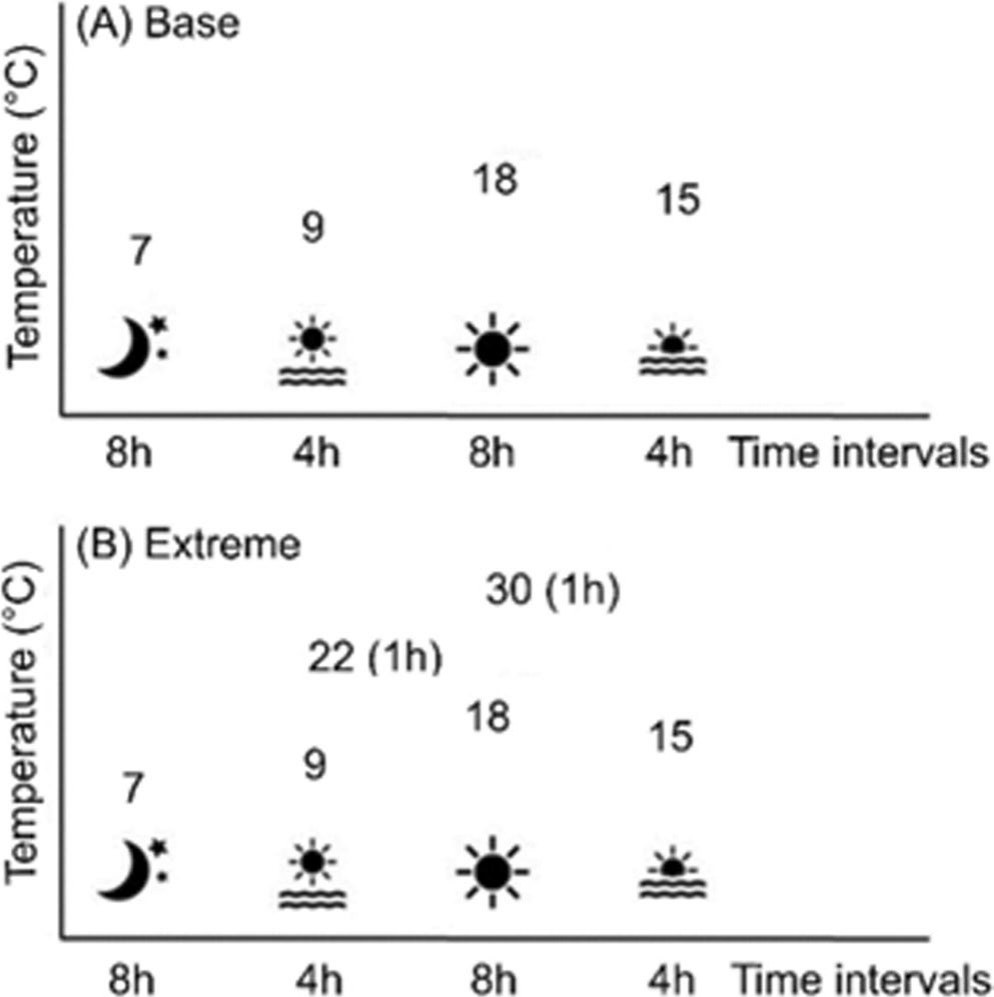
Temperature treatments applied to plants of *V. alpina* in the experimental cultivation. The extreme treatment differed from the base treatment for two 1 h temperature peaks at dawn and during the day.

In 2016, the seeds were sown in Petri Dishes filled with 1% Agar in distilled water + 125 mg/L of Gibberellic Acid (GA_3_) and incubated at 20°C in light‐ and temperature‐controlled incubators. Because the northern and southern populations of *V. alpina* differed in germination timing and dormancy levels (Mondoni et al. 2018), we used GA_3_ to obtain synchronous germination and avoid different developmental stages at the beginning of the growth chamber experiment. The selected amount of GA_3_ proved to synchronize germination without inducing deformities in the seedlings. Soon after radicle emission, 120 seedlings per population were transferred each into a 28 cm^3^ plastic pot with standard soil (Hochmoor substrate, Terflor, Italy) and assigned to two treatments (60 plants per treatment in 20 replicates of three plants each), using a light‐ and temperature‐controlled growth chamber (ClimaCell, MMM Group, Germany) as detailed below. The base treatment simulated the average summer temperature at the warmest southernmost site (PRA) based on temperature measured with a datalogger (MLog5W, Geoprecision, Germany) placed in that site from 30 August 2013 to 24 July 2015. The extreme treatment simulated the temperature values recorded by the same datalogger at PRA during a summer heat wave in July 2016. In particular, the extreme treatment differed from the base treatment for temperature peaks of 22°C at dawn and two one‐hour heat spells (30°C) during the day, applied daily during the whole treatment period (Figure 3). In both treatments, the plants were exposed to a 16 h light/8 h dark photoperiod, 50 μmol m^−2^ s^−1^ photosynthetic photon flux density (PPFD) and received 1 L of tap water three times a week, which guaranteed full soil hydration in the base treatment. The cultivation in the growth chamber lasted 8 weeks for both treatments.

### 2.2 Growth and Ecophysiology

At the end of both cultivation periods, we counted all surviving plants to estimate mortality and measured the height of all surviving plants. The day after the end of the cultivation period, we determined exchanges of CO_2_ and water vapor on a subset of five plants per population, using an open flow system carrying air with ambient CO_2_ pressure (41 ± 1.5 Pa) from the Botanical Garden of Pavia University into an infrared gas analyzer (LCA‐4, ADC Co., Hoddesdon, UK) connected to a factory‐designed small (625 mm^2^) leaf chamber. Illumination was provided by a glasshouse lamp at 450 ± 20 μmol m^−2^ s^−1^ PPFD and ambient temperature (25°C ± 2°C). Each gas‐exchange value was the mean of 3–5 measurements per plant. The gas‐exchange measurements were used for determining area‐based net CO_2_ exchange (net photosynthesis, as μmol CO_2_ m^−2^ s^−1^), stomatal conductance (g_s_, as mol H_2_O m^−2^ s^−1^) and water use efficiency (WUE, as μmol CO_2_ mol^−1^ H_2_O m^−2^ s^−1^). Chlorophyll (chl) a fluorescence was determined in three mature healthy leaves by a portable fluorimeter (OS1‐FL, Opti‐Sciences, Hudson, NH, United States). *F*_m_ was obtained using a saturating pulse of actinic light after 1 min of acclimation, under which conditions *F*_o_ was measured. The ratio *F*_v_/*F*_m_ [with *F*_v_ = (*F*_m_–*F*_o_)] was used to assess the photochemical efficiency of photosystem II as a proxy of stress experienced by the plants during the experimental cultivation. Chlorophyll content was determined (as arbitrary units) on a further subset of 5 plants with a chl content meter (CCM‐200; Opti Sciences, Tyngsboro, MA, USA).

### 2.3 Molecular Analyses

Samples of 30 plants were used for the molecular analyses: eight from SWE, eight from SOB, eight from PRA, and six from NOR. Genomic DNA was isolated from dry leaves using about 20–50 mg of plant material, following the protocol of the genomic DNA purification kit (Thermo Scientific, Waltham, MA, USA). We amplified three noncoding plastid DNA regions: psbD‐trnT spacer, rpL32‐trnL spacer, and trnL‐trnF spacer previously used for testing the variability of these regions in three different angiosperm lineages [*Atropa* vs. *Nicotiana*, *Lotus* vs. *Medicago* and *Saccharum* vs. *Oryza*; Shaw et al. 2007] and for testing phylogeographic variation of *Silene acaulis* (Gussarova et al. 2015). The primers were specifically designated for *V. alpina* from the 10 most similar sequences in GenBank [including *Lychnis alpina* (synonym *V. alpina* ), *Atocion rupestre Silene acaulis*, *S. aprica* , *S. capitata* and other *Silene* species; Table 1]. PCR was performed in a 25‐μL volume with 2 μL template DNA, 7.5 μL distilled H_2_O, 2 μL 25 mM MgCl_2_, 12.5 μL VWR Red Taq DNA Polymerase Master Mix containing the Taq polymerase (VWR Life Science, Milan, Italy) and 0.5 μL (10 pmol/μL) of each primer. The PCR reaction was performed using a BioRad T100 Thermal Cycler as follows: 94°C, 3 min; 40 cycles (94°C, 30 s; annealing temperature, 1 min; 72°C, 1 min); 72°C, 5 min. The annealing temperatures were 55°C for psbD‐trnT, 51°C for rpL32‐trnL, and 56°C for trnL + trnL‐F.

**TABLE 1:** Name and sequence of the primers designed for each DNA region of *V. alpina* chloroplast.

DNA region	Primer name	Primer sequence 5′–3′
psbD‐trnT	Va_psbD_F	CGCGGGTTAAGGTAAGAAAG
	Va_trnT_R	GCGGTCTGTTGAATCMTTAA
rpL32‐trnL	Va_rpL32_F	CACATTTGAAATACGACATC
	Va_trnL_R	GCAAAATCTCTTTTTACYGG
trnL‐trnF	Va_trnL_F	CGAAATCGGTAGACGCTACG
	Va_trnF_R	ATTTGAACTGGTGACACGAG

All samples were sequenced in both directions. The sequencing was carried out by Macrogen Europe with the same primers employed in the PCR reactions. Sequences for each DNA region were aligned using the program Geneious Prime (Biomatters Ltd., Auckland, New Zealand). The obtained sequences were deposited in the INSDC archives through the GenBank platform (Accession numbers from PQ541201 to PQ541212; Table S1). A concatenated data set from the four regions was created and a multiple sequence alignment (MUSCLE Alignment) was generated (Edgar 2004). The total aligned sequence length was 1885 bp: 618 bp for the psbD‐trnT region, 761 bp for the trnL‐trnF spacer, and 506 bp for the rpl32‐trnL region, including gaps and indels. The variation among the studied samples was due to base substitutions.

### 2.4 Statistics

Firstly, we run a one‐way ANOVA on the data of growth‐in‐height in the base treatment with population as the fixed factor. The purpose of this analysis was to assess the effect of provenance on the performance of plants grown under common warm temperature. Secondly, we run a nested two‐way ANOVA on the data of growth‐in‐height with climate nested within geographic area (i.e., northern core area and southern marginal areas). To this aim, we grouped the four populations into two groups, i.e., cold sites (NOR + SOB) vs. warm sites (SWE + PRA). The purpose of the nested ANOVA was to assess effects of biogeographic history, associated with geographic location, vs. putative local adaptation, associated with climate at the location sites. Thirdly, we run six two‐way ANOVAs on growth‐in‐height and five ecophysiological response variables (i.e., net photosynthesis, *g*_s_, WUE, chl content and *F*_v_/*F*_m_) with population and treatment as fixed factors. We also run a generalized logistic regression model with a logit‐link function to determine differences in mortality across treatments, populations, and their interactions. All post hoc comparisons were based on Fisher's LSD tests. The ANOVAs were performed using the package Statistica (v. 13.5. TIBCO Software Inc., Palo Alto, CA, USA). The generalized logistic regression was performed using the SPSS package.

Phylogenetic relationships among *V. alpina* haplotypes were analyzed with the program POPART version 1.7 (Leigh and Bryant 2015). A Minimum Spanning Network was selected as the phylogenetic inference method (Bandelt et al. 1999).

## 3 Results

### 3.1 Growth and Ecophysiology

**FIGURE 4:**
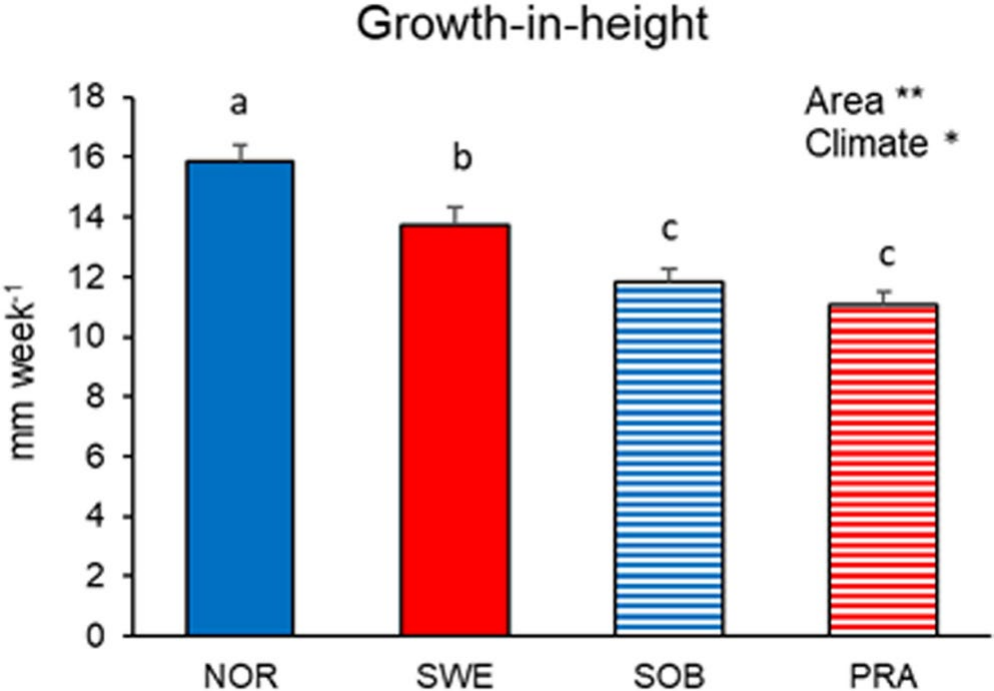
Mean (+1 SE) weekly growth of four *V. alpina* populations cultivated for 8 weeks in a growth chamber at 16 h light/8 h dark photoperiod and temperature conditions simulating the average summer temperature at the southernmost warmest stand (PRA). The means followed by different letters significantly (*p* < 0.05) differed based on Fisher's LSD tests following a one‐way ANOVA. Significance of differences obtained by a two‐way ANOVA (climate nested within geographic area) are summarized by asterisks (**p < 0.01; *p < 0.05). Full columns for the populations in the northern core area; hatched columns for the populations in the southern marginal area. Blue columns for the populations in cold sites; red columns for the populations in warm sites.

**FIGURE 5:**
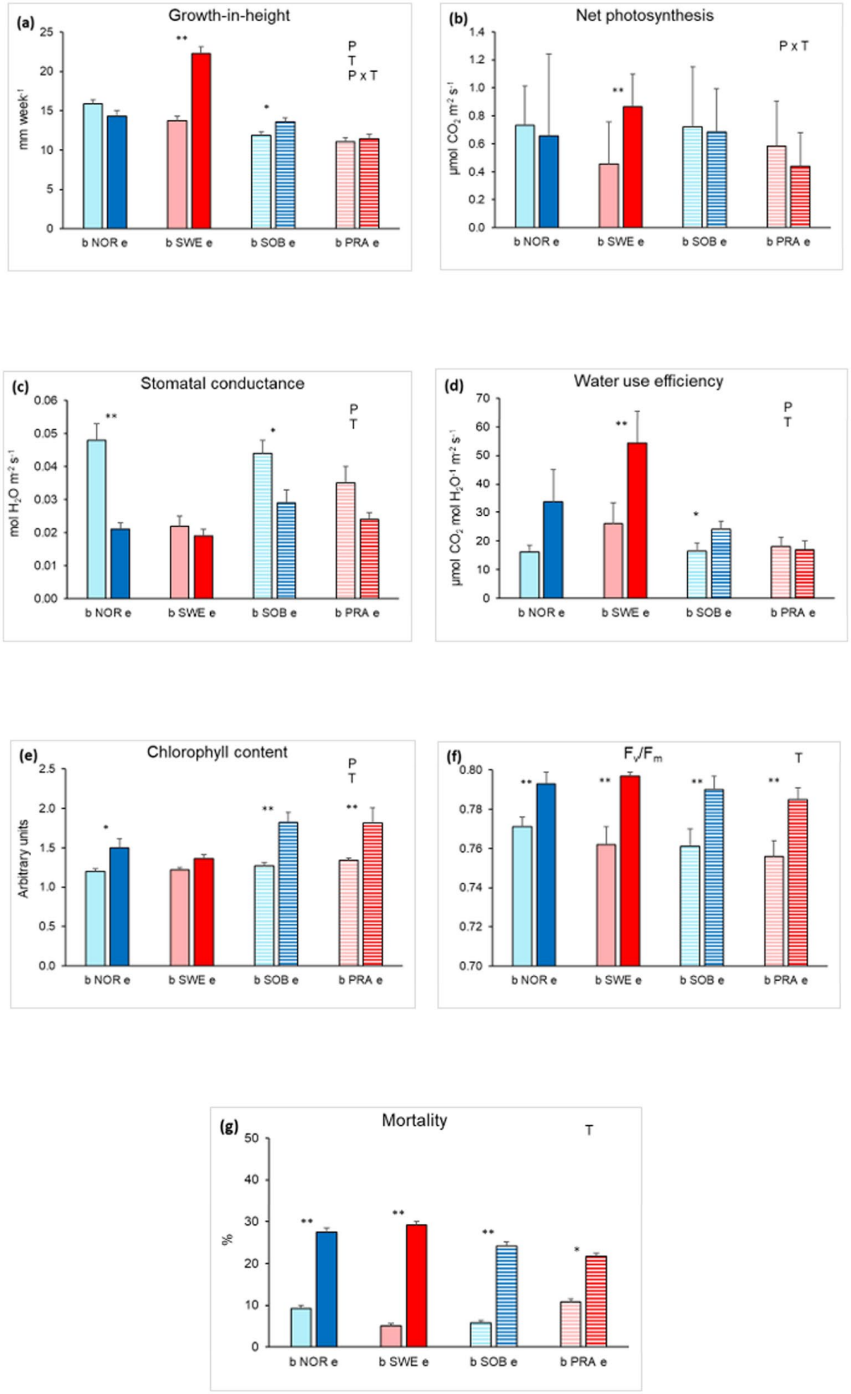
Mean (+1 SE) weekly growth (panel a), net photosynthesis (panel b), stomatal conductance (panel c), water use efficiency (panel d), chlorophyll content (panel e), *F*_v_/*F*_m_ (panel f) and mortality (panel g) of four *V. alpina* populations cultivated for 8 weeks in a growth chamber at: Base (b) treatment (16 h light/8 h dark photoperiod and temperature conditions simulating the average summer temperature at the southernmost warmest stand PRA); extreme (e) treatment (the same conditions as in the base treatment with 2 day‐temperature peaks of 22°C at dawn and 30°C during the day, respectively). Significant (*p* < 0.05) effects of population (P), treatment (T) and their interaction (P × T) resulting from two‐way ANOVAs are shown in the upper right part of the panels. Significance of differences between treatments for each population are summarized by asterisks (**p < 0.01; *p < 0.05). Full columns for the populations in the northern core area; hatched columns for the populations in the southern marginal area. Blue columns for the populations in cold sites; red columns for the populations in warm sites (lighter colors for the base treatment, and darker colors for the extreme treatment).

The growth rate in the base treatment was highest for NOR, followed by SWE, and lowest for SOB and PRA. The growth rate differed both between geographic areas, with higher values in the northern area, and between site climates, with higher values in cold sites. However, the difference was stronger when the populations were grouped by geographic area than by climate (Figure 4). When comparing growth rates of the four populations in the two treatments, there were significant effects of both population and treatment. However, the growth rate differed strongly between treatments for SWE, and to a much a lesser extent for SOB. Both presented higher growth rates in the extreme treatment than in the base treatment (Figure 5a). Net photosynthesis did not present significant main effects either among populations or between treatments, but the net photosynthetic rate was higher for the SWE plants grown in the extreme treatment than in the base treatment, as shown by a significant population × treatment interaction (Figure 5b). The plants grown in the extreme treatment had lower stomatal conductance, higher water use efficiency, and higher chl content compared to the base treatment, though the patterns of differences between treatments for these response variables were not consistent among populations (Figure 5c–e). Similar to net photosynthesis, WUE was higher in the extreme treatment than in the base treatment for SWE and to a lesser extent for SOB. The *F*_v_/*F*_m_ was high (> 0.75) in both treatments (Figure 5f). However, there was a significant effect of treatment because of higher mean *F*_v_/*F*_m_ values in the extreme treatment. The *F*_v_/*F*_m_ did not differ among populations, with no significant treatment × population interaction (Figure 5f). Mortality was significantly higher in the extreme treatment, but it was not affected by population or by treatment × population interaction (Figure 5g). Five variables presented significant effects of treatment when comparing the base vs. extreme response, i.e. growth‐in‐height, *g*_s_, WUE, chl content, and *F*_v_/*F*_m_ (Figure 5).

### 3.2 Molecular and Phylogenetic Analyses

**FIGURE 6:**
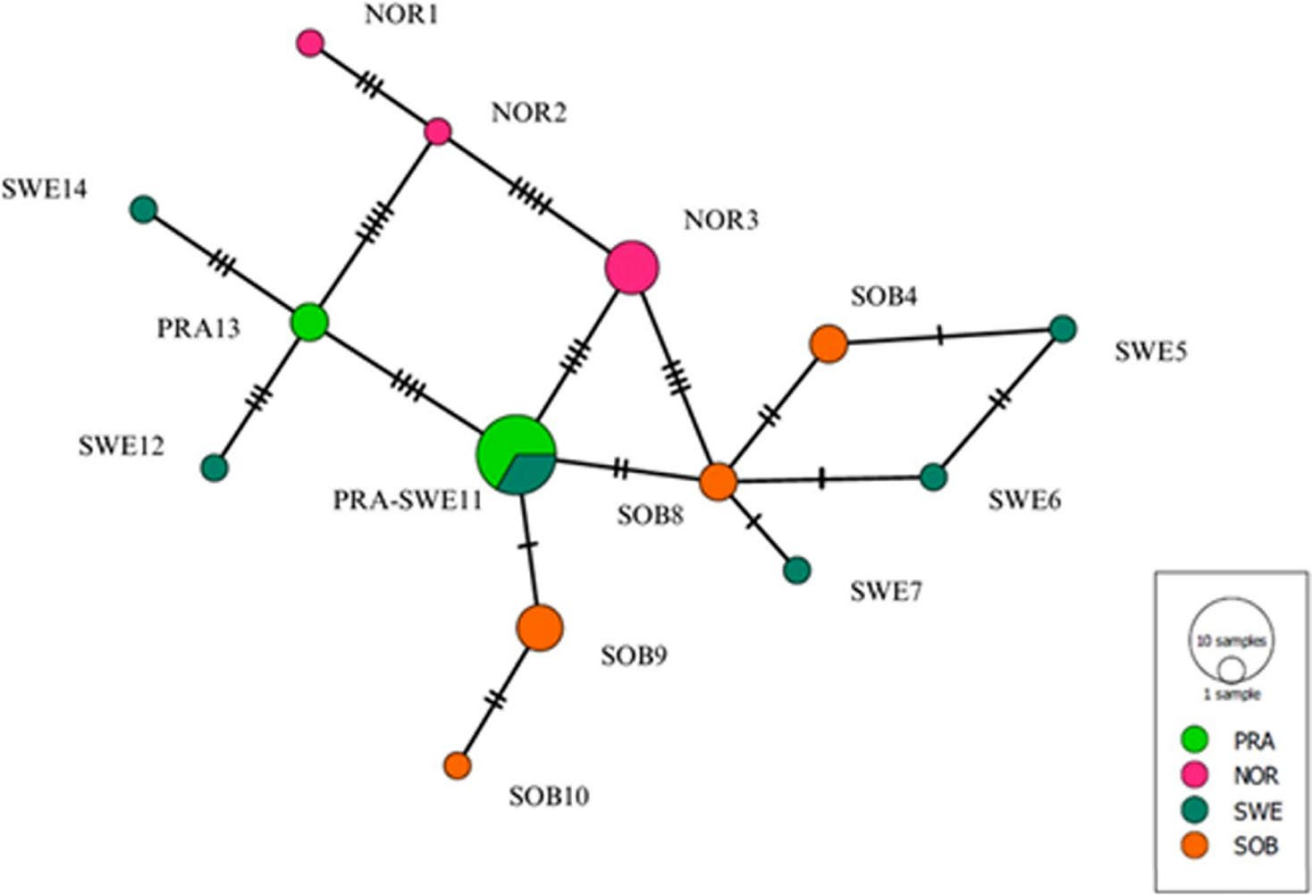
Minimum Spanning Network of the *Viscaria alpina* samples (n=30). The labels indicate plastid haplotype number and the population where the haplotype was found. The bars indicate base substitutions.

The phylogenetic network identified fourteen haplotypes in total and multiple haplotypes in each of the four populations (Figure 6). In total, PRA had two haplotypes, SOB had four haplotypes, SWE had six haplotypes, and NOR had three haplotypes. The SWE haplotypes were overall most different among each other (Figure 6). The PRA and the SOB haplotypes were more similar to those of the SWE population than to those of the NOR population. However, the NOR population differed by only 4 and 5 base substitutions from the most similar haplotypes of SOB and PRA, respectively (Figure 6). The PRA population shared one identical haplotype with SWE, whereas SOB differed by one base from the most similar haplotypes of SWE as well as PRA.

## 4 Discussion

The main goal of our study was to assess whether genetic differences, reflecting phylogenetic history, consistently affect the adaptation of northern vs. southern *V. alpina* populations to a warming climate. The measured genetic distances among populations and the resulting structure of the network do not support a strong genetic divergence among the four populations. One haplotype is even shared by PRA and SWE populations. However, our finding was based on neutral chloroplast DNA markers that are independent of selection and evolve at a slower rate than nuclear markers that are instead subject to gene flow. Similar results were obtained by Frajman et al. (2009), who detected very close relationships between *V. alpina* populations in southern and northern Europe, as well as in populations in Canada and Greenland in cpDNA markers (Frajman et al. 2009). Only a few nuclear markers showed divergence in that same study between two groups in Europe that appeared independent from latitude, but rather suggested independent dispersal events from glacial refugia, as for other alpine‐arctic species with disjunct distribution, like *Ranunculus glacialis* (Ronikier et al. 2012). Overall, our results suggest that *V. alpina* populations of similar present‐day geographical distribution may have had different glacial refugia, and their distribution range may thus have differed strongly during the Pleistocene (Brochmann and Brysting 2008). This has profound implications for the putative genetic differentiation among populations.

When grown under common high temperature, *V. alpina* plants from northern populations grew more than plants from southern populations, although the among‐population differences in survival rate were overall poor. We aimed at checking whether differences in growth rate between northern and southern populations were caused by local adaptation. Local adaptation of arctic‐alpine species has been generally evaluated by cultivating plants from thermally differing, geographically more or less distant provenances grown under common temperature (Quan and Wang 2018; Morente‐López et al. 2020). A general finding of those studies was that plants originating from colder sites flower earlier, grow faster, and have higher survival than plants originating from warmer sites when all plants are cultivated in controlled thermal conditions (DeMarche et al. 2018; Valdés et al. 2019). Such a more active development of plants from colder climates has been interpreted as a counter‐gradient historical adaptation to shorter colder growing seasons (Conover and Schultz 1995). A general assumption when addressing species whose distribution ranges span broad latitudinal gradients is that northern populations are overall adapted to colder habitat conditions than southern populations. In our case, the observed pattern did not reflect local adaptation to current climate conditions because mean temperatures at the original location sites considerably differ from each other in both northern and southern areas. So, the observed geographic differences in plant growth could be determined by a better capacity of northern populations of *V. alpina* , a rather drought‐sensitive species (Nagy 2013), to cope with reduced water availability (Abeli et al. 2015). This can be related to lower precipitation at the northern sites and/or by the poor water‐holding capacity of the shallow soils that usually characterize the pioneer habitats colonized by northern *V. alpina* populations (Nagy 2013). Our finding is in line with similar patterns observed in several arctic‐alpine species whose northern high‐latitude populations have optimum niches in drier conditions than the southern mid‐latitude populations (Wasof et al. 2015).

Despite the overall good tolerance to the heat spells exhibited by *V. alpina* plants, we did observe differences among populations in the level of phenotypic plasticity towards the simulated heat wave. Such differences among populations were principally due to different water acquisition capabilities (Chaves et al. 2002) which result in different WUEs at a given level of stomatal opening (Medrano et al. 2009). The significant increase in net photosynthesis and WUE under heat spells shown by SWE (Figure 5b) resulted in a simultaneous growth stimulation (Figure 5a), consistent with the overall effects of climate warming on plants (Wang et al. 2018; Song et al. 2019). Phenotypic plasticity can evolve and can be selected to cope with variable environments (Ackerly et al. 2000; Richards et al. 2006), promoting population persistence through the development of better adapted traits (Nicotra et al. 2010; Richardson et al. 2017). However, we also found that such a plastic response to warmer growing conditions did not occur in the other populations of *V. alpina* , whose growth was slower and did not vary between treatments. This apparent lack of responses may occur in extreme environments, which select so strongly for stress adaptations that populations evolve decreased plasticity, maintaining traits better adapted to an extreme environmental disturbance (Matesanz et al. 2010). Furthermore, slow growth frequently occurs under limited water availability, allowing plants to better conserve water and to reduce evapotranspiration (Basu et al. 2016; White et al. 2023). This may explain why the populations showing the lowest growth rate (PRA) also showed the lowest mortality in response to heat spells (Figure 5g). Contrary to growth performance, the heat spells negatively affected the survival of the plants exposed to the extreme treatment. Interestingly, Marchand et al. (2005) found increased rates of mortality in spite of increased photosynthetic performance in three species exposed to experimentally induced heat spells at a high Arctic tundra site. All in all, our results support the findings of several studies reporting detrimental effects of warming on alpine and arctic‐alpine species when air temperature was increased by 4°C–8°C above the site average, only when warming was associated with drought (Hernández‐Fuentes et al. 2015; De Boeck et al. 2016; Berauer et al. 2019; Notarnicola et al. 2021). Notably, the highest phenotypic plasticity coincided with the highest number of different haplotypes in the SWE population. There is much evidence that genetically diverse populations of plants inhabiting cold to temperate regions possess high capacity to respond to environmental changes. High phenotypic plasticity has been observed in North American populations of the arctic‐alpine cushion species *Silene acaulis* (Gussarova et al. 2015; DeMarche et al. 2018). Populations of *Heterotheca brandegeei*, a North American chasmophyte, presented high levels of phenotypic plasticity in the response of WUE to compound effects of heat and drought, which may buffer *H. brandegeei* from the negative impacts of climate change (Winkler et al. 2019). Conversely, Wickander et al. (2021) did not observe any relationship between genetic variation and phenotypic plasticity in response to high temperatures in populations of *Persicaria vivipara* , an arctic‐alpine species mostly propagating clonally, with low levels of sexual reproduction. On the other hand, populations of *Argyroxiphium sandwicense* subsp. *macrocephalum*, a Hawaiian mountain plant, lack any genetic control of phenotypic plasticity, although this species presented an overall plastic response, in terms of improved WUE, to a heating and drought treatment (Krushelnycky et al. 2020).

After exposure for 8 weeks to a daily heat spell of 30°C, the plants of all populations performed well in terms of growth performance. This supports the results of several studies reporting increased growth rates of arctic and alpine species exposed to high temperatures (Arft et al. 1999; Abeli et al. 2012; Fazlioglu and Wan 2021). To our knowledge, heat tolerance has never been tested so far in *V. alpina* (but see White et al. 2023). However, previous studies reported critical leaf temperatures > 40°C for several arctic and arctic‐alpine species (Buchner et al. 2017; O'Sullivan et al. 2017). There is evidence that alpine plants can experience leaf temperatures up to 13°C higher than air temperature (Neuner et al. 2000) when absorbing irradiance at PPFD > 1000 μmol m^−2^ s^−1^. The leaf temperature of rosette plants can rise up to 30°C above air temperature at 2 m height above the ground on clear summer days (Larcher et al. 2010). However, our plants were grown at lower irradiance, and net CO_2_ exchange was also measured at light levels about half that of the aforementioned threshold, i.e., about 500 μmol m^−2^ s^−1^. Even if we did not measure leaf temperatures in our experiment, it is extremely unlikely that the temperature in the foliar tissues reached critical levels because the *F*_v_/*F*_m_ values were always close to optimum (Goltsev et al. 2012). In fact, the *F*_v_/*F*_m_ in plant tissues undergoing heat stress, especially at high irradiance, drops far below 0.7 (Streb et al. 2003; Braun and Neuner 2005; Karadar et al. 2018). Indeed, the *F*_v_/*F*_m_ and the chl content both were higher in the plants of all populations experiencing the heat spells. This suggests that the heat spells did not induce damage to the photosynthetic machinery. On the contrary, the photosynthetic efficiency was even improved by the high‐temperature treatment. Similarly, Marchand et al. (2005) observed better photosynthetic performance in four arctic plant species exposed to a temperature rise of approximately 9°C for 8 days. Our study provided indirect evidence that the extreme treatment reduced water content because of evaporation‐driven decline in available soil water when temperatures were raised without concurrent increases in water addition (Elmendorf et al. 2012). In fact, lower stomatal conductance under the extreme treatment revealed reduced water availability in the plants of all populations that experienced the heat spells.

## 5 Conclusion

We conclude that plants of *V. alpina* populations from different provenances are generally able to cope with scenarios resulting from global warming. Overall, the plant performance under warm temperatures reflects adaptation to reduced water availability. Phenotypic plasticity enables the adjustment of the photosynthetic efficiency of *V. alpina* plants experiencing heat spells, and such a response is higher in populations possessing higher phylogeographic variation and experiencing warmer temperatures. However, exposure to heat spells associated with drought is detrimental to plants from all populations because of strongly increased mortality.

## Author Contributions

**Lisa Brancaleoni:** data curation (equal), investigation (equal), writing – original draft (lead), writing – review and editing (equal). **Renato Gerdol:** investigation (equal), writing – original draft (equal), writing – review and editing (lead). **Andrea Mondoni:** conceptualization (equal), data curation (equal), investigation (equal), writing – review and editing (supporting). **Simone Orsenigo:** conceptualization (equal), writing – review and editing (supporting). **Lisa Scramoncin:** investigation (equal), writing – review and editing (supporting). **Carla Lambertini:** investigation (supporting), writing – review and editing (supporting). **Chiara Cianferoni:** investigation (equal), writing – review and editing (supporting). **Thomas Abeli:** conceptualization (lead), data curation (equal), writing – original draft (equal), writing – review and editing (equal).

## Conflicts of Interest

The authors declare no conflicts of interest.

## Supporting information


Appendix S1.

## Data Availability

The data on DNA sequencing are available in the GenBank platform (Accession numbers from PQ541201 to PQ541212). The other data is available at: https://data.mendeley.com/drafts/xxr53chmf7.
