# Supplementary material for: Complex Responses to Climate Warming of Arctic‐Alpine Plant Populations From Different Geographic Provenance
Source: Ecol Evol. 2025 Mar 19;15(3):e71146. doi: 10.1002/ece3.71146 (PMC11922575; doi:10.1002/ece3.71146)
Supplement: Supplementary file 1 — Appendix S1. [file ECE3-15-e71146-s001.docx]

SUPPLEMENTARY TABLE

Table 1. GenBank accession numbers

| Sample | DNA Marker | Accession Number |
| --- | --- | --- |
| Seq1_PRA | psbD-trnT intergenic spacer | PQ541201 |
| Seq2_NOR | psbD-trnT intergenic spacer | PQ541202 |
| Seq3_SWE | psbD-trnT intergenic spacer | PQ541203 |
| Seq1_PRA | rpl32-trnL intergenic spacer | PQ541204 |
| Seq2_PRA | rpl32-trnL intergenic spacer | PQ541205 |
| Seq3_NOR | rpl32-trnL intergenic spacer | PQ541206 |
| Seq4_SWE | rpl32-trnL intergenic spacer | PQ541207 |
| Seq5_SOB  Seq1_PRA | rpl32-trnL intergenic spacer | PQ541208 |
|  | trnL-trnF intergenic spacer | PQ541209 |
| Seq2_NOR  Seq3_SWE | trnL-trnF intergenic spacer | PQ541210 |
|  | trnL-trnF intergenic spacer | PQ541211 |
| Seq4_SOB | trnL-trnF intergenic spacer | PQ541212 |
